# Editorial: Exploring immunotherapy targets and strategies for cancer: a multi-omics perspective

**DOI:** 10.3389/fphar.2024.1506444

**Published:** 2024-11-26

**Authors:** Wanchun Nie, Kailin Tang, Lu Xie, Tianyi Qiu

**Affiliations:** ^1^ Institute of Clinical Science, Zhongshan Hospital, Shanghai Institute of Infectious Disease and Biosecurity, Intelligent Medicine Institute, Fudan University, Shanghai, China; ^2^ School of Life Sciences and Technology, Tongji University, Shanghai, China; ^3^ Shanghai Institute for Biomedical and Pharmaceutical Technologies, Shanghai, China

**Keywords:** cancer therapy, pharmacology, anti-cancer mechanism, cancer prognosis, multi-omics

The progression and metastasis of tumors can be mediated by multiple signaling pathways. Currently, therapies targeting these specific tumor-related pathways are a promising approach against cancer. One important pathway in tumor development is epithelial-mesenchymal transition (EMT) (Fontana et al., 2024). During the EMT process, the cells gradually lose their epithelial phenotypes and change to mesenchymal cells, leading to increased migratory and invasive abilities, which contributed to metastasis. Thus, inhibiting EMT is a potential strategy for cancer therapy.

Some cancer therapies focus on specific EMT-regulated genes and related mechanisms. For example, Huang’s study (Huang et al.), the differentially expressed genes (DEGs) in kidney renal clear cell carcinoma (KIRC) samples were screened and found to be enriched in multiple tumor-related pathways, especially EMT. Among all the DEGs, EMT-regulation-related DEGs including SPARC, TMSB10, LGALS1, and VEGFA were reported to be associated with the cancer prognosis, and were applied to construct the prognostic model for KIRC. Moreover, the function of EMT-related genes was validated using transwell migration and invasion assays, which showed that LGALS1 inhibited KIRC cell migration and invasion, suggesting its anti-cancer potential.

In addition to directly regulating EMT, some therapies target mechanisms closely related to EMT. For example, Tumor-associated macrophages (TAMs) can accelerate EMT by secreting cytokines, growth factors, and chemokines, and affect cancer progression, tumor invasion and metastasis (Zhang et al.). What’s more, TAM could impact the EMT process by fostering an inflammatory milieu, enabling tumor immune evasion, and facilitating interactions with other stromal cells. Therapeutic strategies have been developed to disrupt TAM-induced EMT, reduce TAM recruitment, inhibit TAM-secreted factors, and block the signaling pathway involved in TAM-driven EMT.

Vasculogenic mimicry (VM), a novel form of tumor angiogenesis, has also been proven to be correlated with the development of tumors. Multi-omics-based studies of various cancers have elucidated the relationship between VM and EMT, leading to tumor invasiveness and drug resistance (Tang et al.). Tang’s study emphasized that VM shapes an immunosuppressive microenvironment to promote tumor immune evasion (Tang et al.). Inhibiting VM has become a promising therapeutic strategy, with tyrosine kinase inhibitors (TKIs) and anti-vascular endothelial growth factor (VEGF) antibodies widely used in cancer treatment. Su et al. also demonstrated the potential, illustrating that TKIs could directly impact angiogenesis, and restrain TAM to synergistic reinforcing the immune response with immune checkpoint inhibitors (ICIs), leading to synergistic cancer treatment effects (Su et al.).

While these strategies help suppress tumor progression and metastasis, predicting cancer prognosis remains a significant challenge. Considering the tumor heterogeneity, prognostic analysis of each therapy is essential for personal treatment. Prognostic analysis is essential for personalized treatment and can be measured by indicators such as progression-free survival (PFS), overall survival (OS), overall response rate (ORR), immune-related adverse events (irAEs). Evaluating tumor mutation burden, cytotoxic CD8+ T cells, VHL, cytokine levels, gene expression profiles, and PD-L1 expression also aids in prognosis prediction. Shibata et al. identified immune mediators in the plasma for patients who were treated with anti-PD-1 antibody therapy in urothelial carcinoma, and discovered that these factors are significantly associated with clinical outcomes, which proves the conclusion that construct prognosis prediction models based on these soluble immune mediators can be a promising method. Furthermore, many tumor therapies achieve the judgment of prognosis by specific genes associated with prognosis. Huang’s study identified four prognosis-related genes for kidney renal clear cell carcinoma, which could be involved in suppressing the EMT process (Huang et al.). Statistical analysis validated the link between immune mediators and prognostic outcomes, and a novel prognosis prediction method of EMT-related prognostic signature was designed relying on these genes. Another study illustrated the association of VM with the immunosuppressive TME, as well as the EMT, and VM score is verified to be employed as an indicator to predict the effect of immunotherapy (Tang et al.). In conclusion, prognoses differ from various cancer therapies based on different mechanisms, and the construction of effective personalized treatment requires feature analysis of these specific mechanisms. The prognosis-related factors mentioned provide insight not only into the immediate effects of anti-cancer therapies but also their long-term impacts.

In this Research Topic, we focus on the pharmacology of anti-cancer drugs, and there are five studies in this Research Topic. This article can help further understand the pharmacology of anti-cancer drugs, including the principle of cancer treatment and prediction of prognosis.
